# Outcomes of re-irradiation for oral cavity squamous cell carcinoma

**DOI:** 10.1016/j.bj.2021.12.005

**Published:** 2021-12-27

**Authors:** Yen-Chao Chen, Kang-Hsing Fan, Chien-Yu Lin, Chung-Jan Kang, Shiang-Fu Huang, Hung-Ming Wang, Ann-Joy Cheng, Joseph Tung-Chieh Chang

**Affiliations:** a Department of Radiation Oncology, Chang Gung Memorial Hospital at Keelung, Keelung, Taiwan; b Department of Radiation Oncology, New Taipei Municipal TuCheng Hospital, New Taipei City, Taiwan; c Department of Radiation Oncology, Chang Gung Memorial Hospital at LinKou, Taoyuan, Taiwan; d Department of Otorhinolaryngology, Chang Gung Memorial Hospital at LinKou, Taoyuan, Taiwan; e Department of Medical Oncology, Chang Gung Memorial Hospital at LinKou, Taoyuan, Taiwan; f Department of Medical Biotechnology and Laboratory Science, College of Medicine, Chang Gung University, Taoyuan, Taiwan; g College of Medicine, Chang Gung University, Taoyuan, Taiwan

**Keywords:** Re-irradiation, Head and neck cancer, Oral cavity cancer, Squamous cell carcinoma

## Abstract

**Background:**

To predict the outcome of reirradiation (re-RT) for oral cavity squamous cell carcinoma (OSCC).

**Methods:**

Eighty-three patients met the criterion of having previously irradiated OSCC treated via curative intent re-RT for recurrent or new primary OSCC. The exclusion criteria were a suboptimal dose (<45 Gy) for the first RT and palliative intent for the second irradiation. Re-RT was defined as at least 75% volume at second RT after receiving at least 45 Gy at the first RT.

**Results:**

The 2-year locoregional progression-free survival (LRPFS) and overall survival (OS) rates were 20% and 28%. For LRPFS, four predictors were noted through univariate analyses: performance status (PS) (*p* = 0.001), a dose of at least 60 Gy (*p* = 0.001), stage IVB (*p* = 0.020), and surgery before re-RT (*p* = 0.041). In multivariate analyses, only PS (*p* = 0.005) and a dose of at least 60 Gy (*p* = 0.001) remained significant. For OS, PS (*p* = 0.001) and a dose of at least 60 Gy (*p* = 0.042) were still independently associated predictors, but surgery before re-RT became marginally beneficial (*p* = 0.053). For patients with a poor PS (ECOG = 2–3), the 2-year OS was only 4.5%. Twenty-nine percent of the patients experienced severe late complications (≥Grade 3), and 18% had new episodes of osteoradionecrosis during their follow-up.

**Conclusion:**

We identified PS and a re-RT dose ≥60 Gy as predictors for LRPFS and OS. Surgery before re-RT might improve OS. However, the treatment results of re-RT for OSCC were suboptimal. Prospective trials using modern RT techniques, in combination with new therapeutic drugs or radioenhancers, are warranted for improving these dismal outcomes.

**At a glance commentary**

**Scientific background on the subject**

Most reirradiation (re-RT) studies for head and neck cancer in Western series included patients with all anatomical subsites and pathologic subtypes, which might not reveal the real prognosis of re-RT for oral cavity squamous cell carcinoma.

**What this study adds to the field**

We found that the 2-year locoregional progression-free survival and overall survival rates were only 20% and 28%, with the price of 29% of patients sustaining new grade 3 or higher late complications. For patients with a poor performance status (ECOG= 2-3), treatment with a palliative intent should be considered.

## Introduction

Taiwan is in a region with high prevalence of betel quid chewing, and oral cavity squamous cell carcinoma (OSCC) accounts for nearly 50% of head and neck (HN) cancer [1]. Fifty percent of postoperative patients belong to the high-risk group, which requires adjuvant radiotherapy with or without chemotherapy to improve oncologic outcomes [[2], [3], [4], [5], [6]]. Despite the aggressive locoregional treatment, approximately 30% of patients with OSCC experience postradiation locoregional recurrence (LRR). In addition, approximately 80% of patients with OSCC were habituated to betel quid chewing [2]. As a result of field cancerization, 15% of patients with OSCC experienced second primary tumors (SPTs) during their follow-up, and 70% of them were also in the oral cavity (OC) [6].

When feasible, salvage surgery is the treatment of choice for managing both postradiation recurrence or SPTs of HN cancer. For patients with resectable disease, more than 50% experienced LRR in the absence of further adjuvant treatment [7,8]. For patients with unresectable disease or those who refuse surgery, systemic therapy is traditionally considered as the standard of care. However, even the best available regimen provides a median overall survival (OS) shorter than 1 year and offers little chance of cure [[9], [10], [11]]. Therefore, reirradiation (re-RT), with or without concurrent chemotherapy, is the only potentially curative treatment. The Radiation Therapy Oncology Group (RTOG) conducted two similar phase II trials of repeated concurrent chemoradiotherapy and found that a small but substantial percentage (16%–25.9%) of patients could survive at 2 years [12,13] following re-RT.

However, most studies in Western populations have reported outcomes of re-RT for HN cancer with tumors originating from all anatomic subsites and even the nonsquamous subtype, and only 7%–32% of the patients had OC cancer [[14], [15], [16], [17], [18]]. Considering that different anatomic subsites or pathologic subtypes may result in different outcomes [19], we focused on OSCC and reviewed our previous re-RT experience as a benchmark for future clinical studies.

## Methods and materials

This is a retrospective chart-review study. All patients were treated at a tertiary medical center, Chang Gung Memorial Hospital at Linkou, in Taiwan. The inclusion criterion was previously irradiated OSCC treated with curative intent re-RT for a diagnosis of recurrent or new primary OSCC, including isolated neck recurrences (r-T0N + M0). The exclusion criteria were (1) a suboptimal dose (<45 Gy) of the first RT, (2) patients irradiated only on one side who received re-RT for the other side, (3) palliative intent for re-RT, and (4) re-RT for non-OSCC head and neck cancers, such as nasopharyngeal cancer (NPC) and pharyngolaryngeal cancer. These criteria ensured that characteristics were homogeneous within the study group and that the two RT fields had significant overlap. Re-RT was defined as at least 75% volume at second RT after receiving at least 45 Gy at the first RT. Patients were coded as having recurrent disease if the retreated primary tumor was located at the same subsite of the OC, or if they had lymph node recurrence without an SPT. If a 2-cm separation of normal mucosa existed, or the diagnostic interval was >3 years, then the tumor was coded as SPT. All patients received a restaging work-up according to the standard guidelines at that time, and their stages and recurrence/SPT classifications were reconfirmed by our HN tumor board.

Toxicity was graded according to the Common Toxicity Criteria, version 3.0. The endpoints for this study were locoregional progression-free survival (LRPFS) and OS, calculated from the first day of re-RT by using the Kaplan–Meier method. Univariate analysis (UVA) and multivariate analysis (MVA) were performed using the log-rank and Cox regression methods with stepwise selection, respectively. A significance level of 0.1 was chosen for inclusion of variables into MVA. Hazard ratio (HR), 95% confidence interval (CI) and *p* values were calculated, and values of *p* < 0.05 were considered statistically significant.

### Ethics statement

This study was approved by the Institutional Review Board of our hospital (approval number: 201801575B0). Informed consent was not required due to the retrospective nature of the study.

## Results

### Patients

From December 1999 to March 2010, 83 patients met the criteria of this retrospective chart review study. Of the patients included in this study, 95% were male, and the median age of the patients was 48 years (range, 32–79). All patients had histologically confirmed SCC, and most cases were well-differentiated or moderately differentiated cancers. The most common subsite of OSCC was the buccal mucosa (40%), followed by tumors in the tongue (30%). Seventy percent of the patients were classified as having a recurrence and 30% were classified as having SPTs. The majority of the patients had a betel quid chewing habit (83%), advanced disease (stage IV = 77%), and a good performance status (PS; Eastern Cooperative Oncology Group [ECOG] = 0–1, 73%). The patient characteristics are detailed in Table 1.

**Table 1:** Characteristics of oral cavity squamous cell carcinoma patients with reirradiation.

Characteristics	n	Percentage or range
Total patients	83	100%
Gender		
Men	79	95%
Women	4	5%
Median age	48	32–79
ECOG PS		
0–1	61	73%
2–3	22	27%
The 1st RT primary OSCC sites		
Buccal	33	40%
Tongue	25	30%
Retromolar	10	12%
Gum	10	12%
Hard palate	3	4%
Mouth floor	2	2%
Lip	0	0%
The re-RT irradiated sites		
Oral cavity + -neck	58	70%
Neck only (T0N + M0)	25	30%
Recurrence	58	70%
New primary cancer from oral cavity	25	30%
Stage at re-RT (AJCC 7th ed)		
I	2	2%
II	9	11%
III	8	10%
IVA	34	41%
IVB	30	36%
Squamous differentiation at 1st RT		
Well	37	44%
Moderate	40	48%
Poor	3	4%
Not mentioned	3	4%
Squamous differentiation at re-RT		
Well	20	24%
Moderate	29	35%
Poor	4	5%
Not mentioned	30	36%
Number of cancer events (R + P)		
2	43	52%
3–7	40	48%
Family cancer history	12	15%
Habit(s)		
Smoking	66	80%
Drinking	58	70%
Betel quid chewing	69	83%
Having three	47	57%
None	5	6%

Abbreviations: OSCC: oral cavity squamous cell carcinoma; RT: radiotherapy; re-RT: reirradiation; ECOG PS: Eastern Cooperative Oncology Group Performance Status; AJCC 7th ed: American Joint Committee on Cancer Staging Manual 7th edition; R + P: Recurrence event(s) + New primary cancer event(s).

### Treatments

The median time to re-RT was 15 months, and the median dose of the first RT and re-RT was 64 Gy (range, 59.4–72 Gy) and 60 Gy (range, 28–80 Gy), respectively. Seventy-six patients (92%) received concurrent chemotherapy at re-RT; most of the regimens were cisplatin or methotrexate based, but two patients received a Taxol-based therapy. Regarding the re-RT technique, 59 patients (71%) received intensity modulated radiotherapy (IMRT) or intensity modulated arc therapy, and 24 patients (29%) received conventional or 3D conformal radiotherapy. Because this is not a prospective study, the treatment volume varies significantly by deliberations of treating physicians, but usually no elective nodal irradiation nor prophylactic treatment for low-risk clinical target volume was given, in consideration of severe late complications. Thirty-eight patients (46%) underwent immediate surgery prior to re-RT. The treatment fields included the OC with or without the neck for 58 patients (70%); the rest of the patients (30%) received re-RT only to the neck. In addition, 16 patients (19%) experienced additional cancer events and received irradiation a third time [detailed in Table 2].

**Table 2:** Treatment details of 83 patients received curative intent reirradiation.

Treatment	Values
Median time to re-RT (range)	15 months (2–109)
Median dose of 1st RT (range)	64 Gy (59.4–72)
1st RT for adjuvant setting	77 (93%)
Median dose of re-RT (range)	60 Gy (28–80)
Median volume of re-RT^a^ (range)	200 cm^3^ (18–906)
Re-RT technique	
IMRT/IMAT	59 (71%)
3DCRT + IMRT	3 (4%)
3D-CRT	13 (16%)
Conventional	8 (9%)
Chemotherapy during re-RT	76 (92%)
Surgery before re-RT	38 (46%)
3rd RT	16 (19%)

Abbreviations: RT: radiotherapy; re-RT: reirradiation; IMRT: Intensity modulated radiotherapy; IMAT: Intensity modulated arc therapy; 3D-CRT: 3-dimensional conformal radiotherapy.

a Treatment volumes were only available for 68 patients.

### Response rate, failure pattern and cause of death

Including patients who had received surgery, the treatment response rate was 70%. However, 11 patients (13%) had stable disease and 14 patients (17%) had progressive disease within 2 months after re-RT. Overall, 30% of the patients showed a poor response to the re-irradiation. Moreover, the posttreatment tumor response was a very strong predictor for the patients' LRPFS (*p* = 0.000) and OS (*p* = 0.000).

Two-thirds of the patients experienced locoregional failure without distant metastasis. Sixty-three patients died of head and neck cancer which is related to re-RT. Among them, 51 patients (81%) died of locoregional progression, 12 patients (19%) died of distant progression with or without locoregional disease. Five patients died of another new primary malignancy and 2 patients died of non-cancerous causes.

### Acute and late complications

Because the re-RT fields were limited to gross tumors and high-risk areas with adequate margins, most treatments were tolerated. During the follow-up period, 49 patients (59%) experienced new grade 2 or higher late complications, and 24 patients (29%) experienced new grade 3 or higher late complications. Fifteen patients (18%) had new episodes of grade 2 or higher osteoradionecrosis after re-RT [Table 3]. Besides, we did not find that larger treatment volume at re-RT (≥200 cm^3^or not, *p* = 0.18; ≥300 cm^3^ or not, *p* = 0.21) and higher accumulated dose (≥130 Gy or not, *p* = 0.25) correlated with the incidence of new grade 3 or higher late complications.

**Table 3:** Patients with new grade 2 or higher late complications after receiving reirradiation.

Grade and type of complication	Number of patients
Grade 2 complications^a^	
Xerostomia	7
Trismus	10
Dysphagia	8
Neck fibrosis	6
Hearing impairment	3
Osteoradionecrosis	5
Grade 3 complications^b^	
Trismus	2
Dysphagia	5
Neck fibrosis	3
Metal Plate exposure	3
Soft tissue necrosis/fistula	9
Osteoradionecrosis	7
Grade 4 complications	
Soft tissue necrosis/fistula	2
Carotid Pseudoaneurysm	1
Osteoradionecrosis	3

a 49 patients (59%) experienced new grade 2 or higher late complications.

b 24 patients (29%) experienced new grade 3 or higher late complications.

### Survival endpoints and potential prognostic factors

**Fig. 1:**
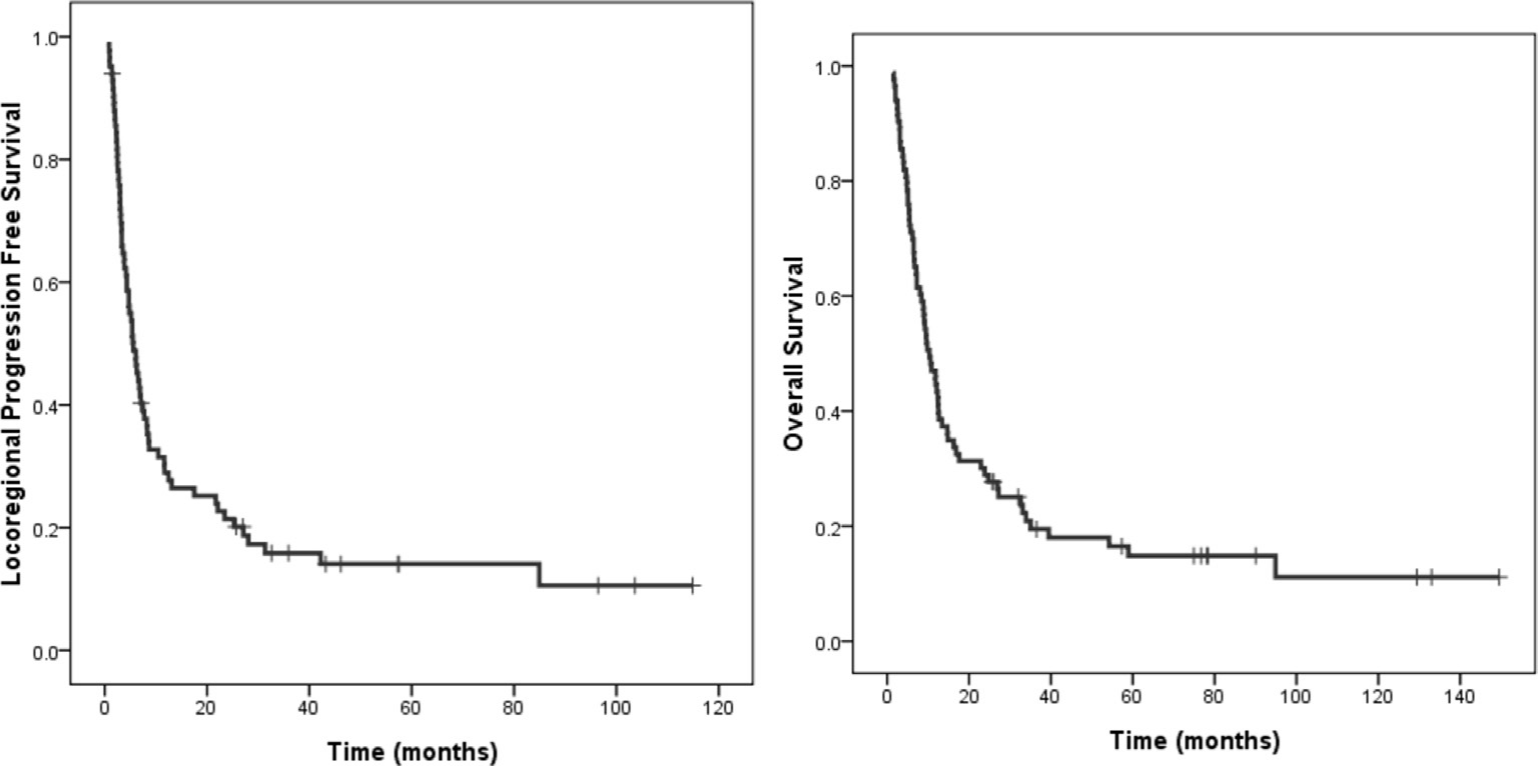
Locoregional progression free survival and overall survival.

**Fig. 2:**
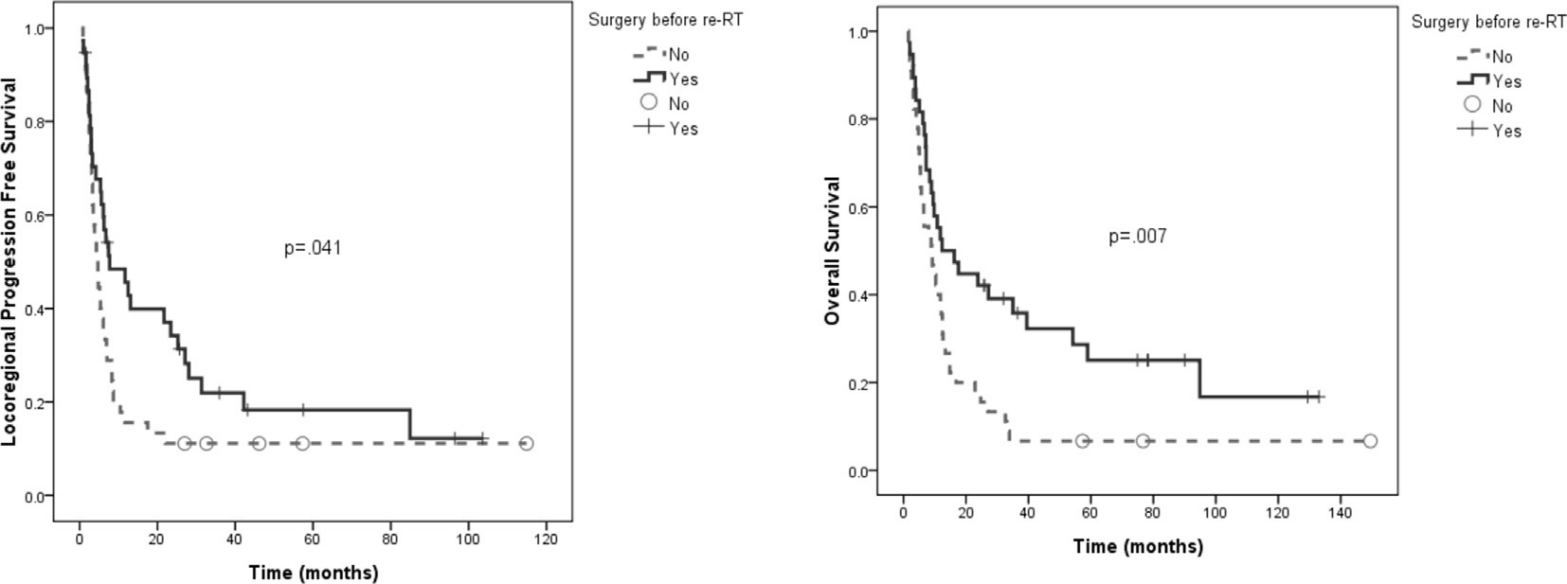
Locoregional progression free survival and overall survival by surgery. Patients who had undergone surgery before re-RT had better locoregional progression free survival (*p* = 0.041) and overall survival (*p* = 0.007) in univariate analysis.

The 2-year LRPFS and OS rates were 20% and 28%, respectively [Fig. 1]. For LRPFS, four pretreatment predictors were noted in univariate analyses: PS (*p* = 0.001), a dose of at least 60 Gy (*p* = 0.001), stage IVB (*p* = 0.020), and surgery before re-RT (*p* = 0.041) [Fig. 2]. In the multivariate analysis, only PS (*p* = 0.005) and a dose of at least 60 Gy (*p* = 0.001) remained significant. The results for univariate analyses of OS were the same as those of LRPFS. For multivariate analysis of OS, PS (*p* = 0.001) and a dose of at least 60 Gy (*p* = 0.042) were significant predictors, but surgery before re-RT became marginally beneficial (*p* = 0.053) [Table 4, Table 5]. There was no significant survival difference in LRPFS or OS after categorizing the subsites of primary tumor at re-RT into tongue, buccal mucosa and others.

**Table 4:** Univariate analyses of predictor factors for locoregional progression-free survival and overall survival.

Factors		N	LRPFS			OS		
			Median (M)	95% CI	*p* value	Median (M)	95% CI	*p* value
Poor ECOG PS	2,3	22	3.0	2.0–4.0	0.001	5.1	3.6–6.6	0.000
	0,1	61	6.7	5.2–8.2		12.5	9.4–15.6	
Stage IVB	yes	30	3.8	2.3–5.3	0.020	6.4	5.0–7.9	0.002
	no	53	7.0			12.3	7.7–16.9	
Surgery before re-RT	yes	38	7.7	0.5–14.9	0.041	12.3	2.1–22.6	0.007
	no	45	4.7	3.4–6.0		9.0	5.1–13.0	
Dose ≥60 Gy	yes	61	6.9	5.2–8.6	0.001	12.1	8.6–15.6	0.043
	no	22	3.0	1.8–4.2		5.7	1.8–4.2	
Tumor in first RT high-dose area	yes	46	5.4	3.8–6.9	0.342	9.5	8.0–11.0	0.167
	no	37	6.7	1.6–11.9		12.5	2.1–22.9	
Age ≥60	yes	13	5.5	4.0–7.0	0.789	9.8	6.0–13.6	0.334
	no	70	6.1	3.9–8.2		10.6	7.4–13.7	
Time to re-RT <10 months	yes	35	5.2	2.5–7.9	0.431	9.5	6.0–12.9	0.992
	no	48	5.6	3.9–7.2		10.6	7.0–14.2	
A + B + C	yes	47	6.1	4.1–8.0	0.933	9.5	6.9–12.1	0.872
	no	36	5.5	2.4–8.6		10.6	7.6–13.6	
Family cancer history	yes	12	6.2	3.0–9.4	0.278	11.8	2.7–20.9	0.217
	no	71	5.5	3.7–7.2		9.8	6.6–13.0	
Recurrent disease	yes	58	4.7	2.8–6.6	0.147	9.0	5.1–12.9	0.081
	no	25	8.2	4.8–11.6		12.5	3.3–21.7	
Cancer events ≥3	yes	40	6.2	3.9–8.6	0.697	10.8	6.9–14.7	0.202
	no	43	5.4	2.7–8.1		9.5	5.2–13.8	
Full course IMRT	yes	61	5.6	4.4–6.7	0.140	9.8	6.8–12.8	0.348
	no	22	4.3	0.0–15.2		10.3	0.0–21.5	

Abbreviations: LRPFS: locoregional progression-free survival; OS: overall survival; ECOG PS: Eastern Cooperative Oncology Group Performance Status; re-RT: re-irradiation; A + B + C: alcohol + betel nut + cigarette; IMRT: Intensity modulated radiotherapy.

**Table 5:** Multivariate analyses of prognostic factors for locoregional progression-free survival and overall survival.

		N	LRPFS			OS		
			HR	95% CI	*p* value	HR	95% CI	*p* value
Poor ECOG PS	23	22	2.044	1.081–3.867	0.028	2.652	1.511–4.654	0.0007
	01	61						
Dose ≥60 Gy	yes	61	0.468	0.261–0.838	0.011	0.542	0.316–0.933	0.027
	No	22						
Surgery before re-RT	Yes	38	0.649	0.369–1.144	0.135	0.588	0.344–1.007	0.053
	no	45						

Abbreviations: LRPFS: locoregional progression-free survival; OS: overall survival; ECOG PS: Eastern Cooperative Oncology Group Performance Status; re-RT: re-irradiation.

### Group stratification for prognosis

**Fig. 3:**
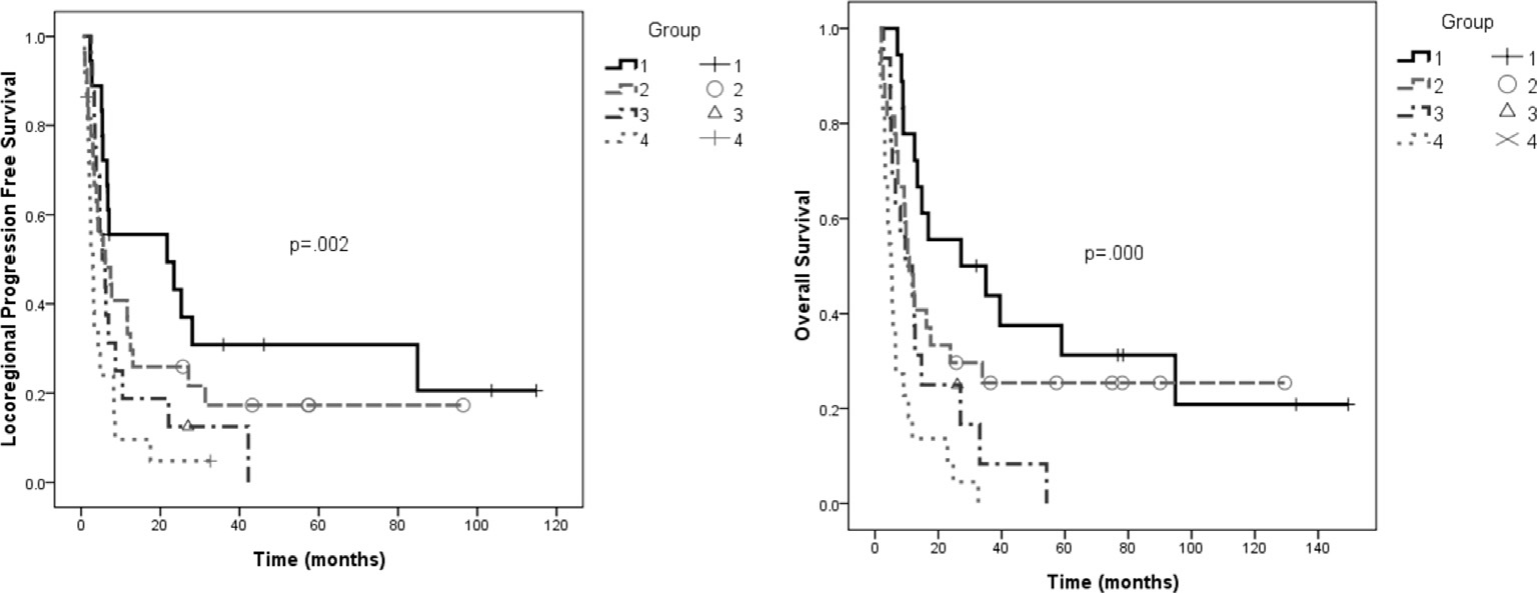
Locoregional progression free survival and overall survival by groups. Group 4 (ECOG = 2–3) had the worst locoregional progression free survival and overall survival. Group 1 (ECOG = 0–1 and stage I–III); Group 2 (ECOG = 0–1 and stage IVA); Group 3 (ECOG = 0–1 and stage IVB).

According to the results of univariate and multivariate analyses for LRPFS and OS, we stratified the patients into four prognostic groups: Group 1: ECOG = 0–1 and stage I–III, n = 18; Group 2: ECOG = 0–1 and stage IVA, n = 27; Group 3: ECOG = 0–1 and stage IVB, n = 16; Group 4: ECOG = 2–3, n = 22. Fourteen of 30 stage IVB patients had a poor PS (ECOG = 2–3), whereas only 8 of 53 stage I to IVA patients had a poor PS (Pearson chi-square test, *p* = 0.002). Group 4 patients with ECOG = 2–3 had the shortest median LRPFS and OS (3.0 and 5.0 months, respectively) [Fig. 3].

## Discussion

Tumor recurrence and SPTs arising within a previously irradiated HN region represent a difficult clinical scenario for the following reasons: (1) survival of tumor cells after definitive RT or CCRT usually implies that they were radioresistant with high malignant potential [11,20]; (2) postradiation changes in normal and tumor tissues, such as decreasing microvascular density and hypoxia [21,22], could put patients at risk of sustaining more unhealed wounds after salvage surgery, developing more tissue necrosis after re-RT, or experiencing poor drug penetration with systemic therapy; and (3) when planning re-RT as a part of salvage treatment, radiation volumes and doses are usually reduced to avoid unnecessary complications, but the accumulated doses remain sufficiently high to cause unneglectable late effects. All of the aforementioned factors contribute to suboptimal outcomes of re-RT with high rates of treatment-related toxicity. Our low 2-year LRPFS and OS rates (20% and 28%, respectively) and new episodes of grade 3 or higher late complications occurring in 30% of the patients may be attributable to these factors.

In the present study, we did not include NPC, which usually has higher salvage 5-year LRPFS and OS rates of approximately 80% and 60%, respectively [[23], [24], [25]]. Lee et al. reported a 2-year OS rate of 37% for HN cancer salvage treatment through re-RT. A subset analysis of their patients with non-NPC SCC showed that the 2-year OS rate for the patients who underwent surgical resection before re-RT was significantly higher than that for patients who did not (36% vs. 12%, *p* = 0.008) [16]; the corresponding figures in our cohort were 41% and 16% (*p* = 0.007). Thus, excluding a subsite with a favorable prognosis, such as the nasopharynx, can worsen the oncologic outcomes relative to those obtained when such subsites are included. Takiar et al. reported re-IMRT results for patients with HN cancer and found that the SCC histologic subtype had a significantly lower OS and locoregional control (LRC) than those of the non-SCC subtype. Moreover, they reported that the OC subsite had unfavorable LRC among patients who underwent surgery and shorter PFS among the patients with definitive re-RT [19]. Although unsatisfactory, the results of our OSCC re-RT were in an acceptable range compared with those of Western studies after we considered the anatomic subsites and pathologic subtypes.

Theoretically, surgery before re-RT removes a majority of the radioresistant clones, thus improving the statistical chance for tumor eradication with re-RT [11]. It is reasonable to assume a more favorable outcome for patients who receive surgery before re-RT. However, in the present study, this factor had statistically significant effects on LRPFS and OS in UVA, but lost their significance in the multivariate analysis, probably because of the influence of PS, disease severity and limited patient numbers.

Some reports state that IMRT can achieve improved LRC or OS [15,16]. However, in the present study, we could not determine the superiority of IMRT in LRPFS or OS, compared with non-IMRT techniques; this could be because not all of our linear accelerators during the treatment time in this series were IMRT capable, and IMRT was administered only to the patients who required it. Most non-IMRT treatments were administered for postoperative neck irradiation or less advanced conditions, in which the dose-delivery technique plays a minor role in optimizing dose distribution. The existence of patient selection bias may obscure the benefits of better dose homogeneity and target coverage offered by the intensity modulation technique.

As compared to SPTs, recurrent disease is usually believed to be more radioresistant because tumor cells were selected from the first RT (11, 20). We observed a 3.5-month shorter median LRPFS (4.7-month vs. 8.2-month, *p* = 0.147) and OS (9.0-month vs. 12.5-month, *p* = 0.081) of recurrent disease without reaching statistical significance. We had a relatively small number of SPTs (n = 25) patients in our study, and the SPTs arose in a postirradiated hypoxic microenvironment, which may reduce the therapeutic effect of re-RT and chemotherapy (21,22). Both factors may contribute to the nonstatistical significance observed.

Another crucial re-RT prognostic indicator frequently mentioned by other investigators is the time interval between the two radiation treatments. The shorter the interval is, the higher the probability that tumors are radioresistant. The RTOG 9610 trial noted statistically improved survival for patients who received re-RT >1 year from the prior RT compared with patients who received re-RT <1 year after prior RT [12]. However, in the present study, the time interval was not found to be a prognostic factor, regardless of whether the cutoff point was 6 months, 10 months, or 1 year. The relatively short median interval (15 months) of our OSCC cohort, together with the fact that 70% of the patients belonged to the recurrent group, may have diminished the discrimination ability of radioresistance over time.

The prognostic factors for LRPFS and OS in the current study, as determined through univariate analyses, were PS, a dose of at least 60 Gy, stage IVB, and surgery before re-RT. Half of the stage IVB patients had a poor PS (Pearson chi-square test, *p* = 0.002). Therefore, the impact of stage IVB might be diluted by the poor PS in the multivariate analysis. Tanvetyanon et al. found that comorbidity and pre-existing organ dysfunction are among several crucial prognostic factors for patients undergoing re-RT, and should be considered in treatment decisions [26]. In the present study, we found that PS was a significant predictor for both LRPFS and OS. Therefore, we suggest that PS can be a simple alternative for evaluating comorbidity and pre-existing organ dysfunction when making a re-RT decision. Most radiation oncologists were reluctant to perform full-dose re-RT for recurrent or second primary HN cancers for fear of severe late complications and poor expected outcomes. Therefore, we stratified the patients into four groups and tried to identify which group benefitted the least from aggressive salvage treatment. Group 4 (ECOG = 2–3, n = 22) had the shortest median LRPFS and OS (3.0 and 5.0 months, respectively), and 2-year OS was only 4.5%. Most of their treatments were palliative in nature.

Based on the retrospective nature of this study, the patient selection bias in determining who received surgery, the RT dose-delivery technique, and limited patient numbers, we only can provide an outline of outcomes for OSCC patients who received salvage re-RT. However, because we confined the study population to oral cavity primary and squamous cell carcinoma only, the relatively focused data may offer as a benchmark for future prospective clinical studies for such patients.

## Conclusion

At the price of 29% of patients sustaining new ≥Gr3 late complications, the 2-year LRPFS and OS rates were 20% and 28%, respectively, for patients with OSCC who received salvage re-RT. Among all prognostic factors, PS was the most crucial, and for patients with a poor PS, treatment with a palliative intent should be considered. Future prospective studies using physical or chemical radioenhancers are warranted to improve the dismal outcomes in such radioresistant OSCC patients.

## Funding sources

This work was supported by grants 107-2314-B-182A-062-MY3 from the 10.13039/501100004663Ministry of Science and Technology in Taiwan and CMRPG3G1411∼3 from the 10.13039/100012553Chang Gung Memorial Hospital in Taiwan.

## Conflicts of interest

None.
